# Peer Review of “AI-Driven and Automated Systems for Continuous Oxygen Saturation Monitoring in Long-Term Oxygen Therapy: Systematic Review”

**DOI:** 10.2196/111439

**Published:** 2026-10-07

**Authors:** Nhung H Hoang

**Affiliations:** 1 Kyoto University of Advanced Science, Kyoto, Japan

**Keywords:** long-term oxygen therapy, artificial intelligence, machine learning, oxygen saturation monitoring, pulse oximetry, closed-loop systems, automated oxygen titration, motion artifacts, chronic obstructive pulmonary disease, systematic review

*This is a peer-review report for “AI-Driven and Automated Systems for Continuous Oxygen Saturation Monitoring in Long-Term Oxygen Therapy: Systematic Review.”*

## Round 1 Review

### General Comments

The authors conducted a systematic review [1] on long-term oxygen therapy. While the topic is relevant and potentially valuable, the manuscript in its current form is poorly formatted and difficult to follow. Revision is needed in terms of structure, clarity, and presentation.

### Specific Comments

#### Major Comments

1. The paper does not clearly summarize the key research questions it aims to address, and the answers presented throughout the manuscript are not well organized. A concise paragraph at the end of the Introduction would help readers understand the scope, objectives, and overall structure of the paper before proceeding to the main sections. In addition, the boundaries between the Methods, Results, and Discussion sections are unclear, with content from these sections often mixed together.

2. In the Methods section, the subsections “Literature Search and Article Selection” and “Study Selection and Eligibility Criteria” appear in an illogical order, and parts of the article selection process are repeated across both subsections. Similar organizational issues appear in the Results section with “Overview of Included Studies” and “Study Characteristics,” where content overlaps substantially. Furthermore, in the “Overview” section, the manuscript mainly describes included studies one by one, which limits synthesis and makes it harder for readers to identify broader patterns across the literature.

3. The manuscript also contains several broad claims that are not adequately supported by evidence. For example, statements such as “few studies discussed failure cases, outliers, or conditions under which the system underperformed” and “many used publicly available datasets (eg, PhysioNet) that lacked full clinical context or were limited in demographic diversity” should be supported by specific citations or a clearer summary of which studies these statements refer to.

4. Separating methodologies into AI and non-AI categories seems somewhat arbitrary, particularly when the review is not explicitly framed around AI in long-term oxygen therapy. The rationale for this categorization should be clarified or reconsidered.

#### Minor Comments

5. The manuscript also requires a careful check of formatting consistency. Citation style is currently inconsistent, with some references presented as numbers, while others use the author-year format. For example, the sentence “The models used techniques such as deep neural networks and Gaussian process regression, often trained on datasets curated under controlled environments (Argüello-Prada & Castillo, 2024)” does not match the numbered citation style used elsewhere in the paper. In addition, subsection formatting is inconsistent, as some subtitles are numbered, while others are not. These issues should be revised to improve professionalism and readability.

## References

[1] Kadariya S, Niraula P, Poudel B, Kadariya S (2026). AI-driven and automated systems for continuous oxygen saturation monitoring in long-term oxygen therapy: systematic review. JMIRx Med.

